# Super and Selective Adsorption of Cationic Dyes onto Carboxylate-Modified Passion Fruit Peel Biosorbent

**DOI:** 10.3389/fchem.2021.646492

**Published:** 2021-05-26

**Authors:** Kaiwei Chen, Linlin Du, Peng Gao, Junli Zheng, Yuanli Liu, Hua Lin

**Affiliations:** ^1^ Guangxi Key Laboratory of Environmental Pollution Control Theory and Technology, Collaborative Innovation Center for Water Pollution Control and Water Safety in Karst Area, Guangxi Key Laboratory of Environmental Pollution Control Theory and Technology for Science and Education Combined with Science and Technology Innovation Base, Guilin University of Technology, Guilin, China; ^2^ School of Textiles, Henan University of Engineering, Zhengzhou, China; ^3^ College of Materials Science and Engineering, Guilin University of Technology, Guilin, China

**Keywords:** dye adsorption, methylene blue, methyl violet, passion fruit peel, anhydride modification

## Abstract

The carboxylate-functionalized passion fruit peel (PFPCS) was an efficient and rapid biosorbent for wastewater treatment. The PFPCS exhibited excellent selectivity to the cationic dyes, where the maximum adsorption capacities for methylene blue (MB) and methyl violet (MV) were 1,775.76 mg g^−1^ and 3,756.33 mg g^−1^, respectively. And the adsorption process of MB and MV on PFPCS reached equilibrium within 20 min. Moreover, the adsorption conditions and mechanisms were investigated. The adsorption process was in good agreement with the pseudo-second-order and Langmuir isotherm models. The adsorption mechanism was also proposed to be electrostatic interaction and hydrogen bond. After six cycles of desorption-adsorption, the removal efficient of MB and MV could be kept above 95%. Thus, PFPCS was considered as a highly efficient absorbent for removing cationic dyes from polluted water due to excellent adsorption characteristics, low cost and environmental friendliness.

## Introduction

Over the past decades, dyes are widely used in textile, paper printing, color photography, food, cosmetics, pharmaceuticals, and leather industries. And the discharge of a large amount of dye wastewater into the water body seriously threatens the ecosystem and human beings and has attracted considerable attention worldwide (Afkhami and Moosavi, 2010). Furthermore, most dyes with aromatic rings are hard to be degraded by light, heat, microorganisms, and chemicals and their transformation products are carcinogenic, teratogenic, and mutagenic (Franco et al., 2018). Thus, dye pollutants need to be urgently removed before the discharge of wastewater into the natural environment. Currently, various approaches, including membrane separation Cao et al. (2020), ion exchange Hassan and Carr. (2018), biodegradation Sosa-Martínez et al. (2020), chemical precipitation Shen et al. (2019), and adsorption Kong et al. (2020); Zhao et al. (2020), are used to treat dye wastewater. Among these, adsorption is regarded as the most promising technology owing to its low cost, great selectivity, and ease of operation.

The development of alternative adsorption materials with high adsorption capacity, high selectivity, and easy regeneration is required for practical application. In the present study, a number of nonconventional and low-cost adsorbents based on biopolymers Yang et al. (2021), cellulose Liang et al. (2020), alginate and agricultural wastes, such as garlic peel Asfaram et al. (2014), peanut husk Sadaf and Bhatti. (2014), orange peel Munagapati et al. (2019), peach gum Zhou et al. (2014), and pomelo peel Argun et al. (2014), were exploited for the effective removal of dyes from aqueous solutions. However, unmodified adsorbents obtained from agricultural wastes have the limitations of low adsorption capacity, slow adsorption rate, and poor adsorption selectivity and reusability. Therefore, more attention has been paid to the development of chemically modified adsorbents based on agricultural wastes by treating them with suitable chemicals.

In this study, passion fruit peels were used as raw materials to remove toxic dyes from wastewater. Passion fruit (*Passiflora edulis f. flavicarpa*), which originated in Brazil, is widely cultivated in tropical and subtropical areas. Passion fruits are mainly used for the industrial production of juice and soft drinks (Lima et al., 2020). Its processing generates large amounts of peel waste accounting for more than 50% of the fruit weight (Abboud et al., 2019). These peels are often ignored and thrown away as waste. Passion fruit peel consists of a large amount of pectin, cellulose, hemicellulose, and other polysaccharides (Silva et al., 2012). These components contain many functional groups such as carboxyl and hydroxyl groups, which are beneficial in terms of adsorbing various organic and inorganic pollutants in the solution. Moreover, the surface hydroxyl groups on cellulose can be modified to improve its hydrophilicity as an adsorbent for dye removal (Zhou et al. 2015). In recent years, the use of modified cellulosic materials in biosorption by introducing carboxyl groups into cellulosic materials via anhydride modification has shown a significant enhancement in the adsorption capacity of dyes and heavy metal ions (Gurgel et al., 2008; Hokkanen et al., 2013; Zhou et al., 2015; Hashem et al., 2020). The present study explored the possibility of using the anhydride-modified passion fruit peel as a potential adsorbent for the efficient and selective adsorption of cationic dyes from wastewater. The anhydride-modified passion fruit peel was treated with a saturated NaHCO_3_ solution because the negatively charged carboxylate group could significantly increase its adsorption capacities for cationic dyes from the aqueous solution by electrostatic attraction compared with the carboxylic acid group (Li et al., 2019a).

To the best of our knowledge, there are no studies on modification of passion fruit peel for removing toxic dyes from wastewater. The present study aimed to investigate the modification of passion fruit peel with succinic anhydride under solvent-free conditions and its subsequent treatment with NaHCO_3_ to enhance its adsorption capacity for cationic dyes from aqueous solutions. The effects of various operating parameters, such as pH, contact time, initial pH of solution, and temperature, on the adsorption capacity of the modified passion fruit peel biosorbent (PFPCS) were explored. In addition, the selective adsorption of cationic dyes from a dye mixture and the regeneration property of PFPCS were also studied. Moreover, the isothermal, kinetic, and thermodynamic parameters were examined to understand the adsorption process. The possible mechanism for the adsorption of MB and MV on the surface of PFPCS was proposed.

## Materials and Methods

### Materials

Passion fruit was obtained from a local market (Guilin, China). Methyl violet (C_25_H_30_N_3_Cl), methylene blue (C_16_H_18_N_3_ClS), amaranth (C₂₀H₁₁N₂Na₃O₁₀S₃), mordant black (C_20_H_12_N_3_NaO_7_S), tartrazine (C_16_H_9_N_4_O_9_S_2_Na_3_), and methyl orange (C_14_H_14_N_3_NaO_3_S) of analytical purity were purchased from Shanghai Aladdin Biochemical Technology Co., Ltd. (Shanghai, China). Hydrochloric acid, sodium hydroxide, hydrogen peroxide, acetone, *N*, *N*-dimethylacetamide, and sodium chloride were provided by Xilong Chemical Co., Ltd. (Shantou, China) and used without any further purification. Succinic anhydride was purchased from Sinopharm Chemical Reagent Co., Ltd. (Shanghai, China). Deionized water was prepared with a water purification AXLM1820 system (Chongqing, China).

### Preparation of PFPCS

Passion fruit peel was separated from the fruit, washed with deionized water three times, and dried at 80°C under vacuum for 8–10 h to constant weight. Then, the dried passion fruit peel was pulverized with a wall breaker and separated by a 100-mesh screen, hereafter, abbreviated as passion fruit peel (PFP).

Five grams of PFP sample was mixed with 100 ml of NaOH (2 M) solution, ultrasonicated for 10 min, and then stirred for 4 h at 80°C. After vacuum filtration, the sample was washed with distilled water. The products were treated with 50 ml of NaOH solution (4 wt%) at 50°C, and then 50 ml of hydrogen peroxide was added drop by drop. After continuous stirring for 60 min, the products were filtered, washed with distilled water to neutral pH and then freeze-dried, hereafter, abbreviated as PFPC.

(Supplementary Scheme S1) illustrates the preparation process of PFPCS. The PFPC derivatives containing free carboxylic groups were prepared by reacting PFPC with succinic anhydride under solvent-free condition. The method was described by Vieira et al. (Vieira et al. 2010). Two grams of PFPC, previously dried at 115°C for 2 h under vacuum, was added to 20 g molten anhydride to maintain a PFPC/succinic anhydride ratio of 1:10 and stirred at the anhydride melting temperature for 6 h. Then, the reaction was stopped by adding DMA. The products were obtained by filtering while hot and washing in sequence with DMA, acetone, and distilled water to remove the unreacted anhydride, DMA and by-products. Subsequently, the products were dried in vacuum at 60°C for 24 h, hereafter, abbreviated as PFPCS.

### Degree of Carboxyl Group

The degree of carboxyl group of PFP, PFPC, and PFPCS was determined by reverse titration as described in a previous study (Gurgel et al. 2008). Then, 100 mg of PFP, PFPC, and PFPCS were treated with 100 ml of NaOH (0.01 M) for 2 h under constant stirring. Then, the adsorbent was separated by filtration and 25 ml of the supernatant obtained was titrated with HCl (0.01 M). The experiment was carried out in triplicate. The degree of carboxyl group was calculated using the following formula:

$${\text{C}}_{\text{COOH}}=\;\left[\frac{\left({\text{C}}_{\text{NaOH }}\times \;{\text{V}}_{\text{NaOH}}\right)\;-\;\left(4\times {\text{C}}_{\text{HCl }}\times {\text{ V}}_{\text{HCl}}\right)}{\text{m}}\right],$$

where C_NaOH_ is the concentration of NaOH solution (mol L^−1^), C_HCl_ is the concentration of HCl solution (mol L^−1^), V_NaOH_ is the volume of NaOH solution (L), V_HCl_ is the volume of HCl solution used in the titration of excessive non-reacted base (L) and m is the mass of adsorbent (g).

### Adsorption Experiment

Adsorption of dyes on PFPCS was performed *via* batch adsorption experiments. Typically, the adsorbent was dispersed in the dye solution, and the mixture was stirred at 150 rpm min^−1^. The pH was adjusted using 0.1 M, NaOH, and HCl solutions. The ionic strength of the solution was adjusted with NaCl. A series of dye solutions with different concentrations were prepared, and the absorbance values were first measured at an appropriate wavelength for plotting calibration curves (Liu et al., 2020). Then, the residual dye concentration was analyzed with an ultraviolet-visible light spectrophotometer. All the adsorption experiments were performed in triplicate. The removal rate for dyes (% removal), adsorption capacity at time *t* (*Q*_t_, mg g^−1^), and equilibrium adsorption capacity (*Q*_e_, mg g^−1^) of PFPCS were calculated using the following formula (Ali et al., 2017; Duan et al., 2019):

$$\%\text{Removal}=\;\frac{C_{0}-\;C_{e}}{C_{0}}\times 100,$$

$$Q_{t}=\;\frac{\left(C_{0}-\;C_{t}\right)\;V}{m},$$

$$Q_{e}=\;\frac{\left(C_{0}-\;C_{e}\right)\;V}{m},$$

where *C*_0_ and *C*_e_ are the initial and equilibrium concentrations of dyes in the solution (mg L^−1^), respectively, *C*_t_ is the dye concentration (mg L^−1^) at time *t* (min), *V* (L) is the volume of the dye solution, and *m* (mg) is the weight of PFPCS adsorbent.

### Selective Adsorption of Cationic Dyes From the Dye Mixture

Six groups of the dye mixture, including MB/MO, MB/TTZ, MV/MO, MV/TTZ, MB/MV/MO, and MB/MV/TTZ, were used to evaluate the adsorption selectivity of PFPCS. Typically, 50 mg PFPCS was added to 10 ml of the dye mixture. The initial concentration of cationic and anionic dyes was 0.1 mM. After the adsorption process, the mixture was filtered to determine the dye concentration.

### Regeneration Study

The recyclability of PFPCS was evaluated by using the solvent desorption technique. The dye-adsorbed PFPCS was added into HCl solution (1.0 M) for desorption three times. The mixture was sonicated for 30 min and then adsorbent was collected *via* centrifugation. Finally, the treated adsorbent was dried at 60°C to a constant weight and stored for next use.

### Measurements and Characterizations

FT-IR of samples were recorded on a Thermo Nexus 470FT-IR spectrometer using KBr disk technique in the range of 400–5,000 cm^−1^ (Nicolet Company, MA, United States). Thermo gravimetric analysis (TGA) wascarried out on a Q500 thermogravimeter (TA Company, DE, United States) under nitrogen flow with a heating rate of 20°C min^−1^. The morphologies of Y-PFP were collected on an S-4,800 field-emission scanning electron microscope (HITACHI, Japan). The surface charges of Y-PFP was measured with a Nano ZS90 nanoparticle-size and zeta potential analyzer (Malvern Company, Malvern, United Kingdom) with pH in the range of 2–10. The crystal structure of Y-PFP was recorded using an X’Pert PRO wide-angle X-ray scatterer (Panalytical, Almelo, Netherlands). Ultraviolet (UV) spectra was recored on a Lambda 365 ultraviolet-visible light spectrophotometer (PerkinElmer Co., Ltd., Shanghai, China). The Brunauer-Emmett-Teller (BET) surface area of the sample was measured using a Quantachrome NOVA 1200e Series (Quantachrome, United States) at 77.35 K.

## Results

### Material Characterization

The amounts of carboxyl groups on PFPC and PFPCS were calculated to be 2.85 mmol·g-1 and 6.27 mmol·g-1 by titration, respectively (Table 1). The increase of carboxyl groups confirmed the success of the proposed modification.

**TABLE 1:** Degree of succinylation of the biosorbents.

Biosorbent	C_COOH_ (mmol g^−1^)
PFP	1.55 ± 0.25
PFPC	2.85 ± 0.36
PFPCS	6.27 ± 0.06

**FIGURE 1:**
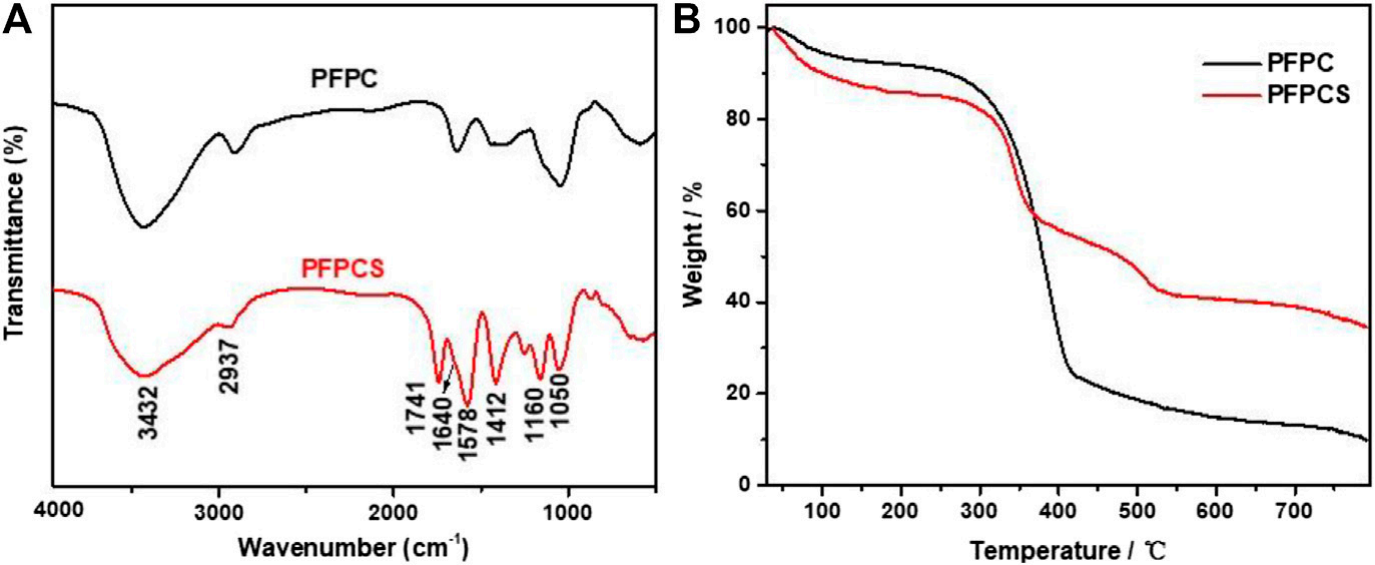
Fourier-transform infrared (FT-IR) curves **(A)** and thermogravimetric analysis (TGA) curves **(B)** for PFPC and PFPCS.

**FIGURE 2:**
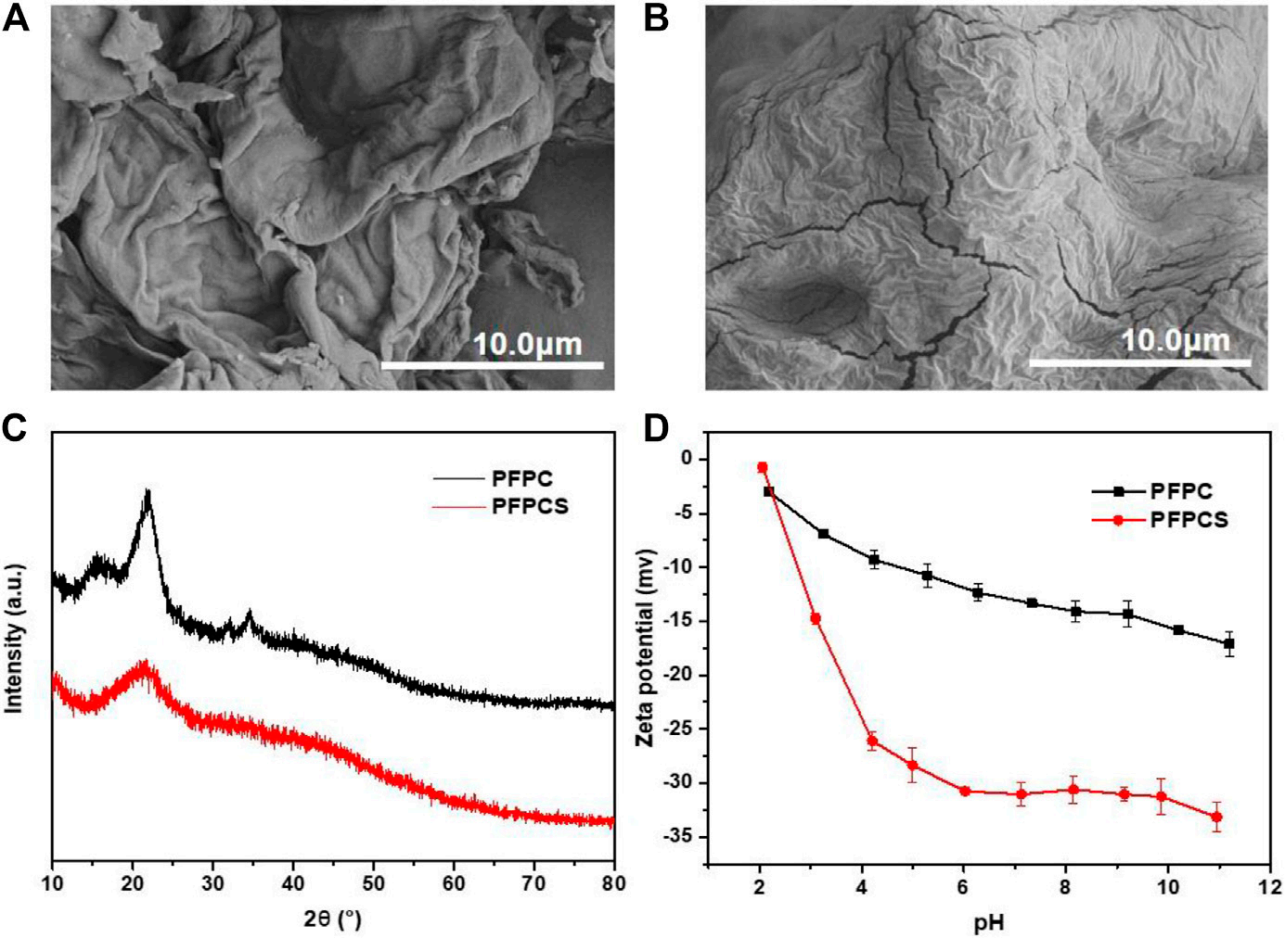
The SEM images of PFPC **(A)** and PFPCS **(B)**, respectively. **(C)** The XRD pattern of PFPC and PFPCS. **(D)** The Zeta potential curves of PFPC and PFPCS.

The FT-IR spectra of PFPC and PFPCS are shown in Figure 1A. The peaks at 3,432 cm^−1^, 2,937 cm^−1^, 1,640 cm^−1^, and 1,050 cm^−1^ were attributed to O-H stretching vibration, C-H stretching of CH_2_ and CH_3_, O-H and C-O-C stretching vibration of cellulose, respectively (Li et al., 2019b; Shen et al., 2019; Xu et al., 2020; Sillkla et al., 2012). And for PFPCS, the new peaks at 1741 cm^−1^, 1,578 cm^−1^, 1,412 cm^−1^, and 1,160 cm^−1^ appeared after anhydride modification which were characteristic peaks of C=O stretching vibration of carboxyl and ester groups, asymmetric and symmetric stretching vibrations of ionic carboxylic groups, and C-O antisymmetric stretching vibration, respectively (Zhang et al., 2010). The results indicated that the succinic anhydride was successfully introduced into the surface of PFPC with a high number of carboxyl groups. Figure 1B shows the thermogravimetric (TG) curves of PFPC and PFPCS. Two-stage weight losses can be observed for PFPC, whereas PFPCS shows three-stage weight losses process. The first stage of weight loss at 30–100°C resulted in moisture evaporation in the samples. The mass loss of PFPC and PFPCS was 5.08 and 9.99%, respectively. In the higher temperature range, thermal decomposition behavior of PFPCS is different from PFPC, which might be attributed to the thermal cleavage of the organic material and the scission of the glucosidic units. In the derivative thermogravimetric (DTG) curves (Supplementary Figure S1), the initial thermal decomposition temperature of the PFPCS is lower than that of PFPC, and a new thermal cleavage peak of PFPCS appears around 390°C. The explanation to these differences could be sought by the broken intermolecular hydrogen bonds and weak intermolecular interaction caused by the modification reaction (Shang et al., 2016). At 800°C, the percentage of the remaining PFPC and PFPCS samples was 9.90 and 34.50%, respectively, which mainly comprised ash and inorganic compounds. The higher residual percentage of PFPCS might be because of a minor level of the inorganic salt of ionic liquid remaining in the modified sample. What’s more, the SEM micrographs of PFPC and PFPCS show in Figure 2. The PFPC and PFPCS exhibited layered structure with rough and wrinkled surface, providing more effective adsorption sites for dye molecules. And the XRD patterns for PFPC and PFPCS are presented in Figure 2C. The PFPC had two intense peaks at 2*θ* of 15.54° and 21.85°, which were the characteristic peaks of cellulose I structure (Zhang et al. 2005). However, the PFPCS had a peak at 2*θ* of 21.44°, indicating that the modification process destroyed the original crystal structure. Besides, chemically modification resulted in a reduced specific surface area and pore volume of PFPC (Supplementary Figure S2). The specific surface area and pore volume of PFPC were 4.403 m^2^ g^−1^ and 0.014 cm^3^ g^−1^, respectively. However, the specific surface area and pore volume of PFPCS were determined to be 1.941 m^2^ g^−1^ and 0.002 cm^3^ g^−1^, respectively. The pores on the surface of PFPC may be blocked after chemical modification, which resulted in a reduced specific surface area and pore volume of PFPCS. And the values of the zeta potential of PFPC and PFPCS were measured at different pH (Figure 2D). The PFPC and PFPCS were negatively charged at pH 3–10, which was attributed to the presence of abundant carboxylic groups (Zhou et al., 2014). The lower zeta potential of PFPCS suggested the succinic anhydride modification introduced a large number of carboxyl groups on the surface of PFPCS, which was beneficial for the adsorption of cationic pollutants (Song et al., 2018).

### Adsorption Characteristics of PFPCS

#### Adsorption Performances of PFPCS Adsorbent for Various Dyes

**FIGURE 3:**
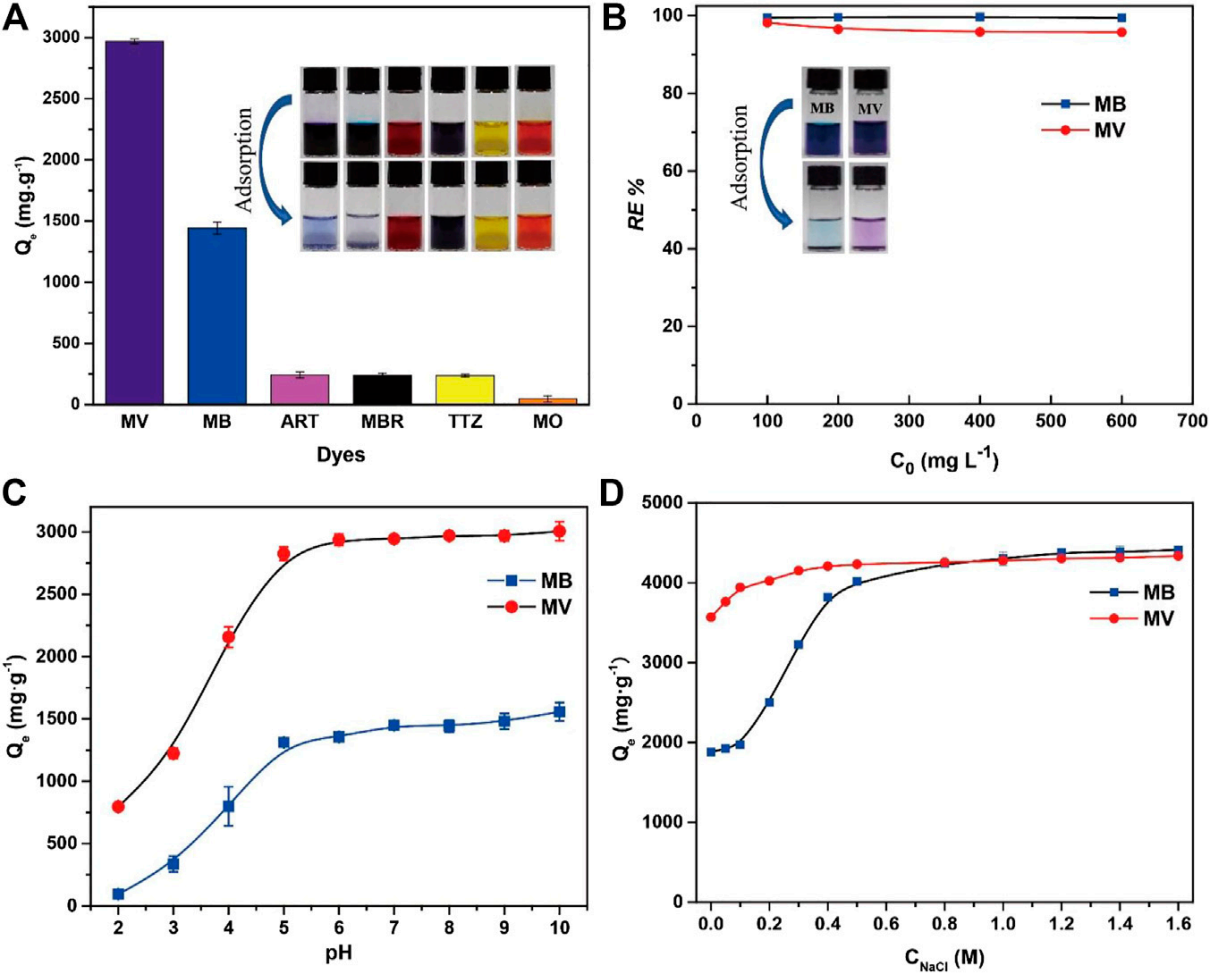
**(A)** Adsorption capacity of PFPCS for different dyes. Insets: photographs of different dyes (1,000 mg L^−1^) before (above) and after (below) adsorption by PFPCS. **(B)** The effect of initial dye concentration on the removal efficiency of PFPCS towards methyl violet (MV) and methylene blue (MB) dyes. Insets: photographs of MB and MV solutions (100 mg L^−1^) before (left) and after (right) adsorption by PFPCS. **(C)** The adsorption performance of PFPCS for MV and MB at different pH. **(D)** Effect of ionic strength on the adsorption of MB and MV by PFPCS at pH = 8 and 25°C.

Six dyes were selected to study the adsorption capacity of PFPCS for different dyes with the initial concentration of 1,000 mg L^−1^. The structure of dyes is shown in (Supplementary Table S1). As shown in Figure 3A, the adsorption capacity for MV reached 2,926.62 mg g^−1^, about 13 times higher than that for anionic dyes (ART, MBR, TTZ, and MO). And the image showed that almost all cationic dyes (MV and MB) were removed by PFPCS, while no distinct adsorption of anionic dyes (ART, MBR, TTZ, and MO) was observed. Thus, the PFPCS exhibited excellent adsorption performance for cationic dyes (MB and MV). Besides, the removal efficiency of PFPCS for MB and MV had only a little decreased (Figure 3B) with increase of initial dye concentration from 100 mg L^−1^ to 600 mg L^−1^. The excellent adsorption performance of PFPCS toward cationic dyes was attributed to the strong interactions between the negatively charged (−COO^−^) and the positively charged functional groups of dyes (such as amino groups). However, the anionic dyes (ART, MBR, TTZ, and MO) contained negatively charged (-SO_3_^−^) with electrostatic repulsion for PFPCS, resulting in poor adsorption performance. And MB and MV were chosen to investigate the adsorption kinetics and isotherms.

#### Effects of pH and Ionic Strength

The degree of ionization, adsorbent surface charge and nature of dye solution were significantly affected by the pH (Mashkoor and Nasar, 2019). Therefore, the influence of solution pH on the adsorption performance of PFPCS was evaluated at the pH range of 2–10 with *C*_0_ of 1,000 mg L^−1^ (Figure 3C). The adsorption capacities of MB and MV increased from 94.11 mg g^−1^ to 1,557.34 mg g^−1^, and 796.11 mg g^−1^–3,006.00 mg g^−1^ with increase of pH, respectively. The low adsorption capacity in an acidic environment was attributed to the adsorption competition between hydrogen ion and cationic dyes for the binding sites on PFPCS (Song et al., 2018). And the carboxyl groups on surface of adsorbent were protonated under acidic conditions, resulting in a decrease of the number of active binding sites for cationic dyes. The surface charge of PFPCS became more negative and the adsorption competition from hydrogen ion gradually declined with the increase of pH. Thus, the enhancement of electrostatic interactions between the negatively charged PFPCS and the cationic dyes led to a remarkable increase of adsorption capacity. Moreover, the adsorption capacity for MB and MV reached a plateau from pH 6 to pH 10. And the pH of industrial wastewater generally has a range of 6–9. Therefore, the optimum pH value was selected as 8.

In addition, the effect of ionic strength on the adsorption capacity is displayed in Figure 3D. The adsorption capacities for MB and MV enhanced with the increase of NaCl concentration and reached the maximum at the concentration of 0.8 M. The phenomenon was attributed to the increase in the dimerization of reactive dyes in solution. The increase of ionic strength suppressed electrostatic repulsion, improving dye aggregation and adsorption capacity (Hu et al., 2013).

#### Adsorption Kinetics

**FIGURE 4:**
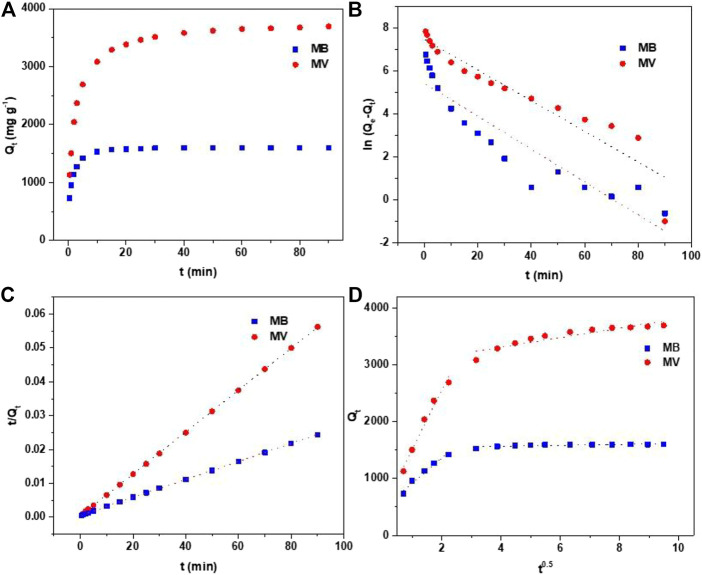
**(A)** Effect of contact time on adsorption of MB and MV by PFPCS at pH = 8 and 25°C. **(B)** Pseudo-first-order kinetic model plots. **(C)** Pseudo-second-order kinetic model plots. **(D)** Intraparticle diffusion model plots.

To further understand the adsorption mechanisms, the effect of adsorption time of PFPCS for MB and MV was investigated with the initial dye concentration of 500 and 1,000 mg L^−1^ at pH 8 and 25°C, respectively. As shown in Figure 4A, the adsorption capacity rapidly increased during the first 10 min and reached the equilibrium within 25 min. The high adsorption rate was attributed to the strong electrostatic attraction between the carboxyl groups of PFPCS and positively charged of cationic dyes, which had obvious advantages in practical applications. The pseudo-first-order, pseudo-second-order and intraparticle diffusion models were used to further investigate the adsorption mechanism (Manjunath and Kumar, 2018; Li et al., 2019a; Cai et al., 2020).

$$\text{Pseudo}-\text{first}-\text{order}\;\text{equation:}\;ln\left(Q_{e}-\;Q_{t}\right)=lnQ_{e}-\;k_{1}t,$$

$$\text{Pseudo}-\text{second}-\text{order}\;\text{equation:}\;\frac{t}{Q_{t}}\;=\;\left(\frac{1}{k_{2}Q_{e}^{2}}\right)+\;\frac{t}{Q_{e}},$$

$$\text{Intrapartical}\;\text{diffusion}\;\text{model}:\;Q_{t}=k_{i}t^{\;0.5}+C_{i},$$

where *k*_1_ (L h^−1^) and *k*_2_ (g (mgh)^−1^) are the pseudo-first-order and pseudo-second-order rate constants of adsorption, respectively. *Q*_t_ (mg g^−1^) is the amount of adsorbed metals at time *t* (min), *Q*_e_ (mg g^−1^) is the equilibrium adsorption capacity, *k*_i_ is the intraparticle diffusion rate constant and *C*_i_ is the intercept of the stage *i* associated with the thickness of the boundary layer.

The linearly fitted lines and parameters of PFPCS for MB and MV adsorption are shown in Figures 4B,C and Table 2. The $Q_{e-cal}$ values based on the pseudo-second-order kinetic model were in good agreement with the experimental data $\left(Q_{e-exp}\right)$. And the adsorption process more accorded with the pseudo-second-order kinetic model with the higher correlation coefficients (*R* ^2^) values (0.9999 for both MV and MB), suggesting that the adsorption process was the chemical adsorption. In addition, the intraparticle diffusion model was described in Figure 4D. The deviation of straight lines from the origin revealed that pore diffusion was not the sole rate-controlling step (Liu et al., 2012; Liu et al., 2016; Manjunath and Kumar, 2018). The adsorption rate was also affected by electrostatic attraction, ion exchange and other factors.

**TABLE 2:** Adsorption kinetic parameters of the adsorption of methylene blue (MB) and methyl violet (MV) onto the PFPCS adsorbent at pH = 8 and 25°C. The initial concentrations of MB and MV were 500 and 1,000 mg L^−1^, respectively, and the contact time is 60 min.

Dye	Q_e-exp_ (mg g^−1^)	Pseudo-second-order			Pseudo-first-order			Intraparticle diffusion		
		t/Q_t_ = 1/K^2^Q_e_^2^ + t/Q_e_			ln (Q_e_– Q_t_) = lnQ_e_ - K_1_t			Q_t_ = K_i_t^0.5^ + C		
		Q_e_-cal (mg g^−1^)	K_2_ (g mg^−1^ min^−1^)	R_2_	Q_e-cal_ (mg g^−1^)	K_1_ (min^−1^)	R_2_	K_i_	C	*R* ^2^
MV	1,600.50	1,613.96	0.0010	0.9999	229.38	0.076	0.8572	72.60	1,095.06	0.5911
MB	3,692.65	3,757.93	0.0001	0.9999	1780.71	0.071	0.9012	243.1	1851.78	0.7470

**FIGURE 5:**
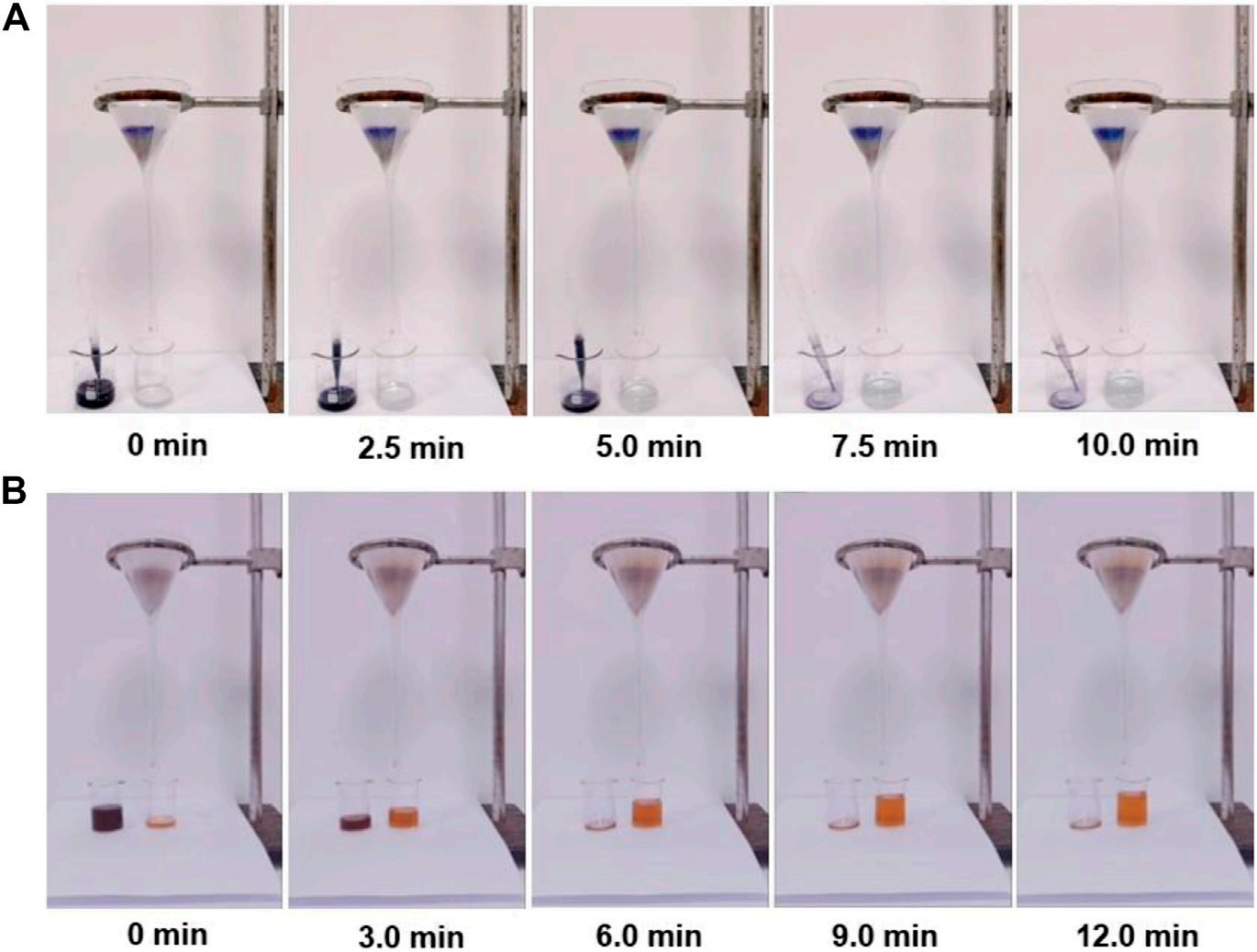
**(A)** Screenshots of adsorption videos based on the addition of 15 ml of MB (1,500 mg L^−1^) solution to passthrough an PFPCS column. **(B)** Screenshots of adsorption videos based on the addition of 20 ml of MV/MO (500 mg L^−1^) solution to pass through an PFPCS column. 100 mg of dried PFPCS was utilized to construct the column.

Considering the practical treatment of dye wastewater, the use of PFPCS powder to construct a column filler for the removal of cationic dyes from an aqueous solution was also investigated. As shown in Figure 5A and Supplementary Video S1 mmc1, colorless water was collected when passing 15 ml of MB solution (1,000 mg L^−1^) through the PFPCS column by gravity, indicating that almost all the MB molecules were intercepted by PFPCS. The result demonstrated that the as-prepared PFPCS had outstanding continuous separating capacity for cationic dyes, thus providing direct evidence for the application potential of PFPCS in continuous dye separation.

### Adsorption Isotherms

**FIGURE 6:**
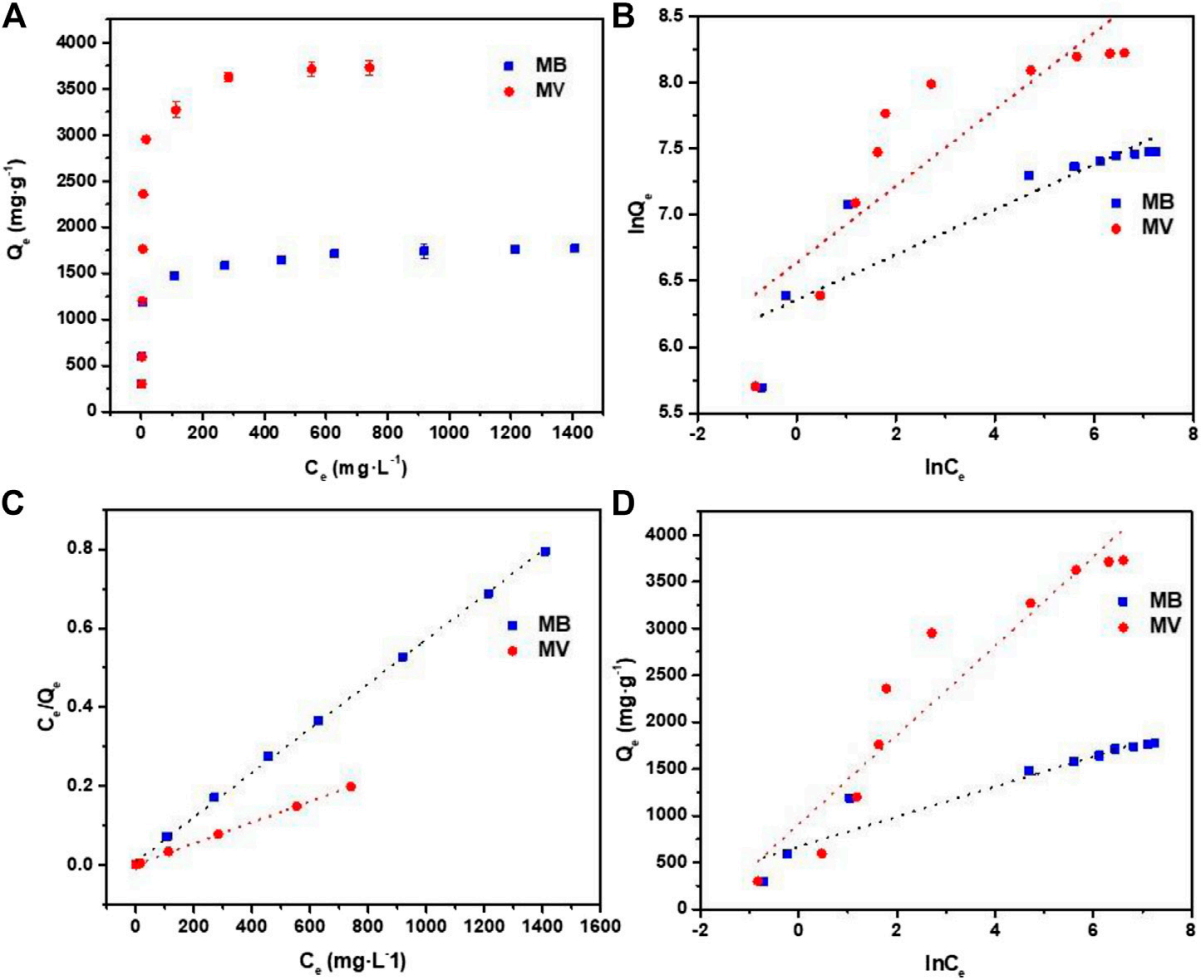
**(A)** Adsorption isotherms for the adsorption of MB and MV by PFPCS at pH = 8 and 25°C. **(B)** Freundlich isotherm model plots. **(C)** Langmuir isotherm model plots. **(D)** Temkin model plots.

Besides, the Figure 6A show the adsorption isotherms to understand the interaction of dye molecules and PFPCS. The adsorption capacity of PFPCS for MB and MV increased gradually with the increase of initial dye concentration and reached an equilibrium at higher concentrations. Because the strong driving force overcame the mass transfer resistance of dye molecules from the aqueous to the solid phase in the adsorption process. And the Langmuir, Freundlich, and Temkin isothermal models were selected to analyze the adsorption data (Huang et al., 2018; Xu et al., 2020; Yao et al., 2020).

$$\text{Langmuir}\;\text{isotherm}:\;\frac{C_{e}}{Q_{e}}=\frac{C_{e}}{Q_{max}}\;+\frac{1}{Q_{max}K_{L}},$$

$$\text{Freundlich}\;\text{isotherm}:\;lnQ_{e}=lnK_{F}\;+\;b_{F}lnC_{e},$$

$$\text{Temkin}\;\text{isotherm}:\;Q_{e}=\;BlnK_{T}\;+\;BlnC_{e},$$

where *Q*_max_ (mg g^−1^) is the maximum adsorption capacity of the adsorbent, *K*_L_ (L mg^−1^) is the Langmuir adsorption constant related to the adsorption energy, *K*_F_ is the Freundlich constant related to the adsorption capacity, *b*_F_ is a constant depicting the adsorption intensity, *B* is the Temkin constant related to the heat of sorption (J mol^−1^), and *K*_T_ is the equilibrium binding constant related to the maximum binding energy (L mg^−1^).

The fitting results and parameters are shown in Figure 6B-D and Table 3. The correlation coefficient (*R* ^2^) of the Langmuir model was 0.9997 and 0.9994 for MB and MV, respectively, which were higher than that of the other two isotherm models. Therefore, the adsorption process agreed well with the Langmuir isothermal model, indicating that the MB and MV adsorption on PFPCS was monolayer and took place at specific adsorption sites (Manjunath and Kumar, 2018). And the essential characteristics of a Langmuir isotherm could be expressed by a dimensionless equilibrium parameter, *R*_L_:

$$R_{L}=\;\frac{1}{1+\;{\text{K}}_{\text{L}}C_{0}},$$

where K_L_ is the Langmuir constant (L mg^−1^) and *C*_0_ is the initial concentration of dye (mg L^−1^). The favorability of adsorption process was considered to be irreversible (*R*_L_ = 0), favorable (1 > *R*_L_> 0), unfavorable (*R*_L_> 1) or linear (*R*_L_ = 1) based on the *R*_L_ value (Song et al., 2018). The *R*_L_ value was in the range of 0.0036–0.1316, implying favorable adsorption of MB and MV dyes on PFPCS. And the maximum adsorption capacity $\left(Q_{max}\right)$ of PFPCS for MB and MV was 1,775.76 mg g^−1^ and 3,756.33 mg g^−1^, respectively, which were higher than that of most of the reported modified adsorbents (Table 4). Therefore, the PFPCS biosorbent had great potential in the removal of MB and MV from aqueous solutions.

**TABLE 3:** Adsorption isotherm parameters for the adsorption of MB and MV onto the PFPCS at pH = 8 and 25°C. The contact time was 60 min.

Isotherm model	Parameters	MB	MV
Langmuir: C_e_/Q_e_ = C_e_/Q_m_ + 1/Q_m_K_L_	Q_m_/(mg g^−1^)	1775.76	3,756.33
	K_L_/(L mg^−1^)	0.066	0.137
	*R* ^2^	0.9993	0.9998
Freundlich: lnQ_e_ = lnK_F_ + b_F_lnC_e_	K_F_/(mg g^−1^)	577.45	764.55
	b_F_	0.170	0.289
	*R* ^2^	0.7748	0.7277
Temkin: Q_e_ = BlnK_T_ + BlnC_e_	K_T_/(L mg^−1^)	65.17	6.72
	B/(KJ^−2^ mol^−2^)	160.34	476.81
	*R* ^2^	0.9087	0.8916

**TABLE 4:** Comparison of adsorption capacity of PFPCS with other adsorbents reported previously.

Adsorbents	Dye types	Q_max_ (mg/g)	References
NbO/g–C_3_N_4_	MB	373.1	Gan et al., 2018
CMC/GOCOOH composite microbeads	MB	180.23	Eltaweil et al., 2020
Modified palygorskite	MB	527.22	Wang et al., 2015
CS-g-PAM	MB	1917	Junlapong et al., 2020
CH–Mt/PANI	MB	111	Minisy et al., 2021
Walnut shell-based activated carbon	MB	400.11	Li et al., 2020
PFPCS	MB	1775.76	Present study
MMT/GO/CoFe2O4	MV	97.26	Foroutan et al., 2020
Gum xanthan/Fe_3_O_4_ based nanocomposite hydrogel	MV	642	(Mittal et al., 2016)
Multi-carboxylic magnetic gel	MV	400	Song et al., 2018
Graphene oxide hydrogel composite	MV	1,052.63	(Makhado et al., 2018)
Dithiocarbamate-grafted star-like polymer	MV	1,239	Liu et al., 2020
Polyacrylamide	MV	1,136	Rahchamani et al., 2011
PFPCS	MV	3,756.33	Present study

#### Adsorption Thermodynamics

In order to further understand the adsorption mechanism, the effect of temperature on the adsorption process of MB and MV was investigated. And the different thermodynamic parameters, such as Gibbs free energy change (∆*G* ^0^, kJ mol^−1^), enthalpy change (∆*H* ^0^, kJ mol^−1^), and entropy change (∆*S* ^0^, J mol^−1^ K^−1^), were calculated according to the Van’t Hoff equation Liu et al.(2020) as follows:

$$ln\left(\frac{Q_{e}}{C_{e}}\right)\;=\;\frac{\Delta S^{0}}{R}\;-\;\frac{\Delta H^{0}}{RT},$$

where *Q*_e_ (mg g^−1^) denotes the amount of dye adsorbed per gram of PFPCS, *C*_e_ (mg L^−1^) represents the equilibrium concentration of dyes, *R* stands for the ideal gas constant (8.314 J mol^−1^ K^−1^), and *T* (K) denotes the reaction temperature.

**FIGURE 7:**
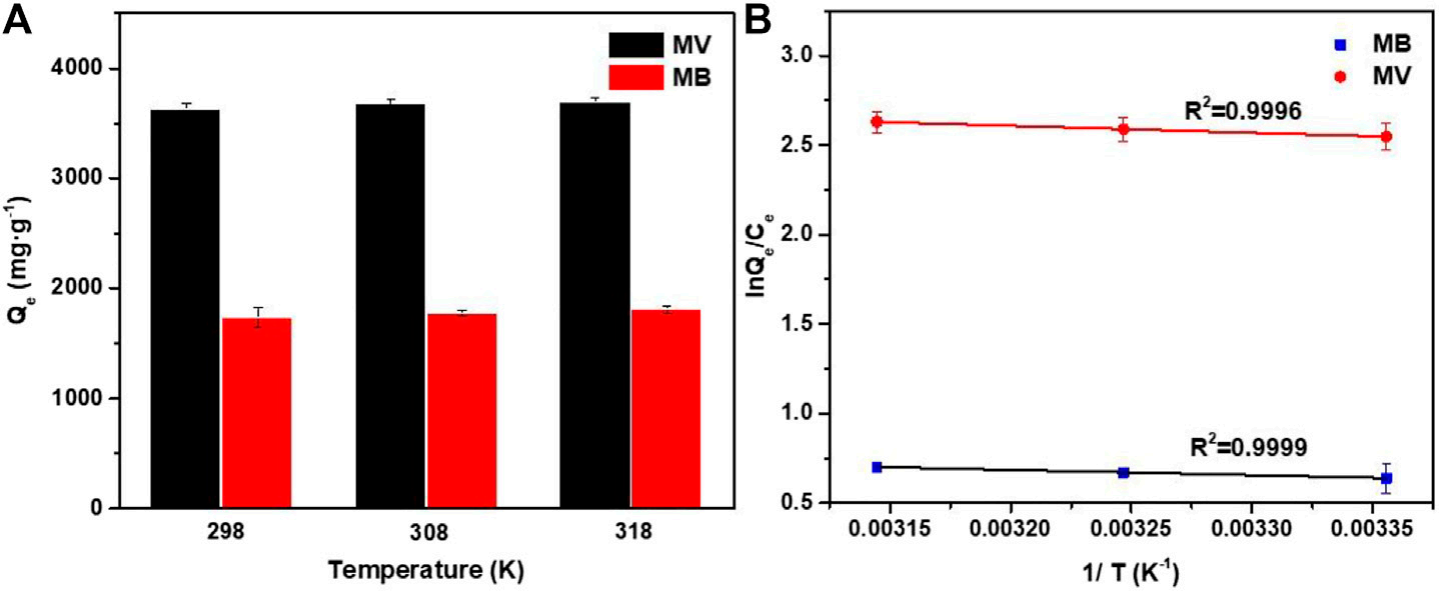
**(A)** Adsorption capacities of PFPCS for MB and MV at different temperature with initial dye concentration of 1,500 mg L^−1^ (pH = 8). **(B)** Plots of lnQe/Ce against 1/T for the adsorption of MB and MV onto the PFPCS.

As shown in Figure 7, the adsorption capacity of PFPCS for MB and MV increased with an increase of temperature. Plotting ln (*Q*_e_/*C*_e_) against 1/*T* gave straight lines with a high *R* ^2^ value of 0.9999 and 0.9996 for MB and MV (Figure 7B), respectively, indicating that the adsorption data were in good agreement with the Van’t Hoff equation (Liu et al., 2020). And ∆*G* ^0^ was calculated using the following formula:

$$\Delta G^{0}\;=\;\Delta H^{0}\;-\;T\Delta S^{0}$$

The calculated thermodynamic parameters are shown in Table 5. The positive ∆*H* ^0^ suggested the endothermic nature of MB and MV adsorption onto PFPCS. In addition, the ∆*G* ^0^ values were negative and decreased from −1.57 and −6.30 kJ mol^−1^ to −1.84 and −6.94 kJ mol^−1^ with an increase in the temperature from 298 to 318 K, respectively. This result suggested that the adsorption of MB and MV onto PFPCS was spontaneous and favorable at higher temperatures, which was consistent with the experimental results shown in Figure 7A. Besides, the positive values of ∆*S* ^0^ for MB and MV adsorption on PFPCS suggested the increase in randomness at the solid/solution interface after MB adsorption onto PFPCS surfaces (Bhatti et al., 2017).

**TABLE 5:** Thermodynamic parameters for the adsorption of MB and MV onto the PFPCS at pH = 8. The initial dye concentration is 1,500 mg L^−1^ and the contact time is 60 min.

Dye	∆H^0^ (KJ mol^−1^)	∆S^0^ (J mol^−1^ K^−1^)	∆G^0^ (kJ mol^−1^)		
			298 K	308 K	318 K
MB	2.42	13.44	−1.57	−1.71	−1.84
MV	3.23	32.03	−6.30	−6.62	−6.94

#### Selective Adsorption Experiments and Regeneration Study

**FIGURE 8:**
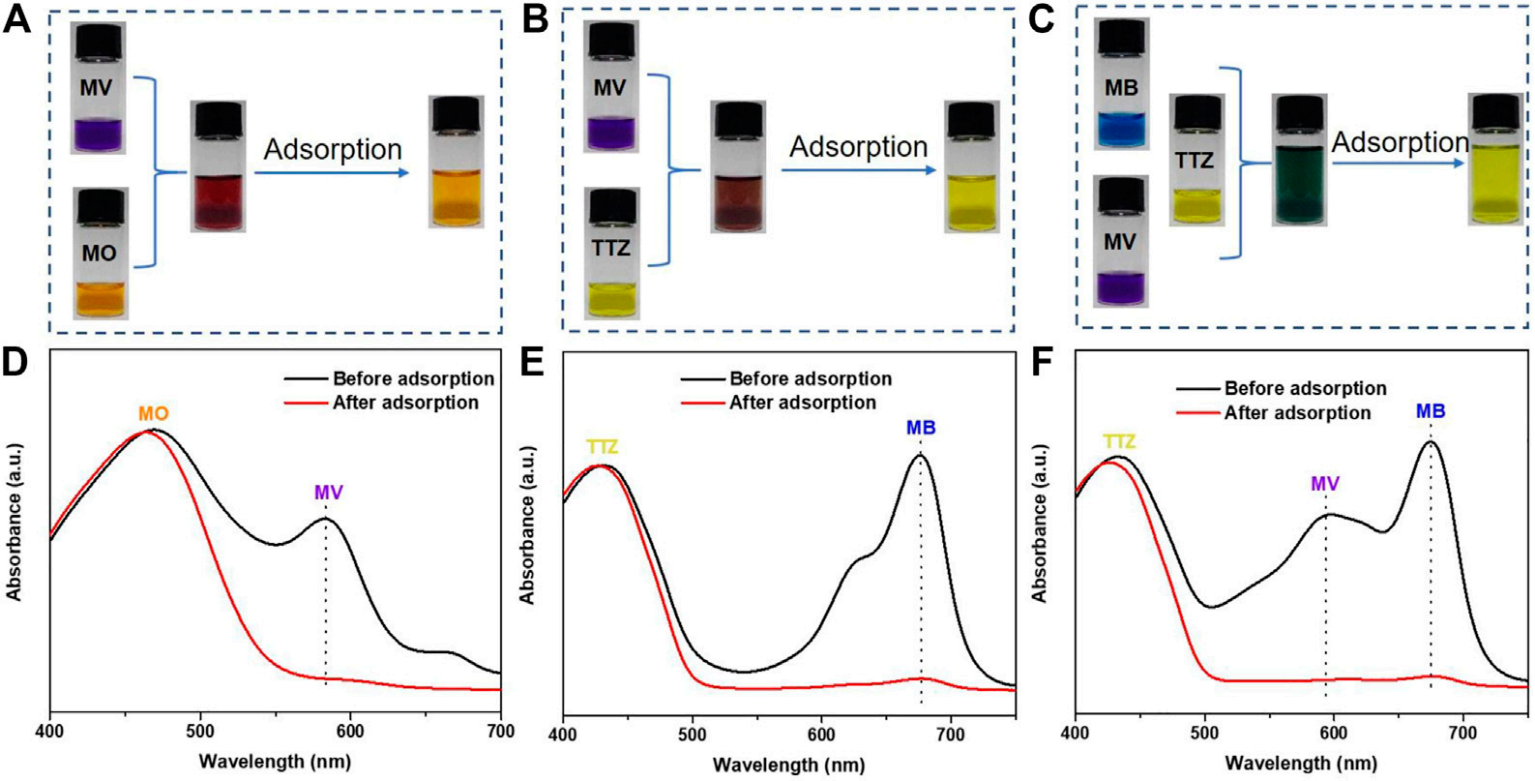
Photographs of the dye mixture of MV/MO **(A)**, MV/TTZ **(B)**, and MB/MV/TTZ **(C)** before and after adding PFPCS for 60 min at pH = 8. The initial dye concentration in the mixture is 0.1 mM. The corresponding absorption spectra of the dye mixture of MV/MO **(D)**, MV/TTZ **(E)**, and MB/MV/TTZ **(F)** before and after adsorption by PFPCS.

Currently, it is increasingly desirable from the perspective of resource recovery to develop a highly selective adsorbent for dye separation from practical dye wastewater. The adsorption effect of PFPCS for different dye mixture (MB/MO, MV/TTZ, MB/MV/MO, MB/TTZ, MV/MO, and MB/MV/TTZ) was shown in Figure 8 and Supplementary Figure S3. The color of cationic dyes (MB and MV) faded after adsorption, and the color of the mixture was close to the anionic dye. And as shown in Figures 8D–F and (Supplementary Figure S3D-F), each dye mixture exhibited two or three independently intense absorption bands before adsorption. After the addition of PFPCS, the intensity of the absorption bands corresponding to cationic dyes (MB or MV) decreased significantly. While that of the anionic dye (MO or TTZ) change weakly, indicating that PFPCS had excellent selectivity for cationic dyes (MB and MV). Moreover, the PFPCS column was also used to study the selective adsorption potential for cationic dyes. For example, the collected effluent showed a distinct orange color related to the MO dye when passing 20 ml of the mixture of MV/MO dye (500 mg L^−1^) through the PFPCS column by gravity (Figure 5B and Supplementary Video S2 mmc2), suggesting that the MV dye was selectively adsorbed by PFPCS. Therefore, PFPCS was potentially used to treat cationic dye–containing wastewater.

**FIGURE 9:**
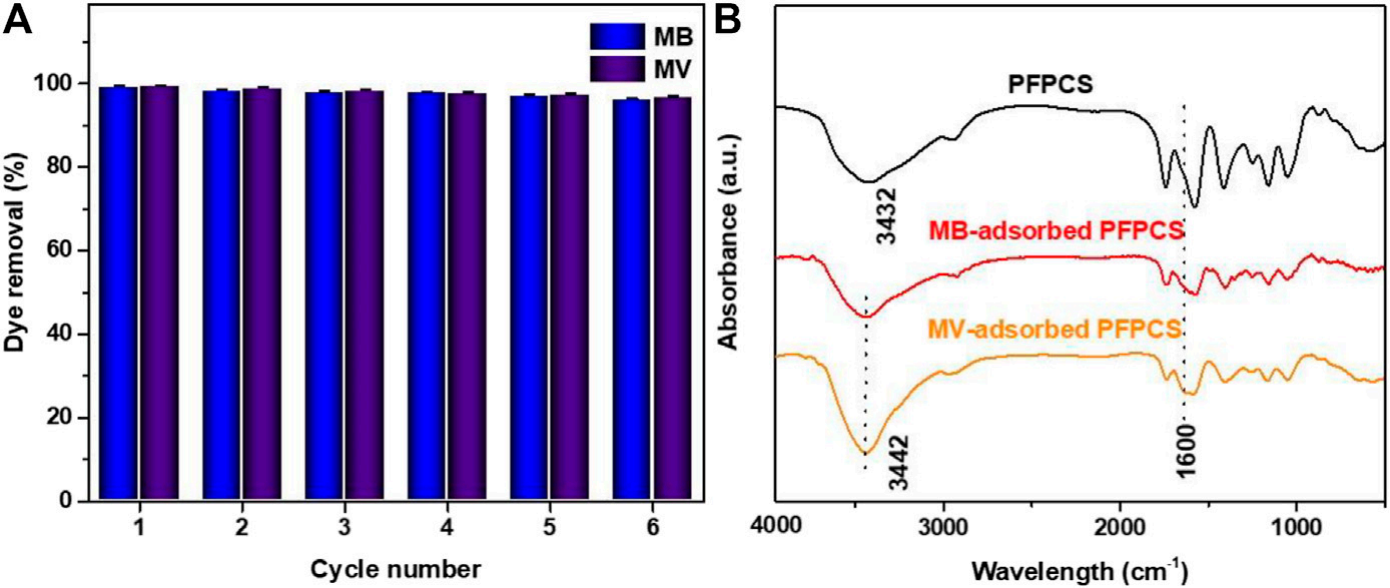
**(A)** Removal rate of the PFPCS in six successive cycles of desorption-adsorption. **(B)** FTIR spectra of PFPCS before and after adsorption of MB and MV dyes.

In practice, the regeneration is also a key index of the performance of adsorbents. Considering the poor adsorption capacity of PFPCS at low pH, the regeneration experiments were carried out in a 1 M HCl solution. As shown in Figure 9A, the removal rate of PFPCS for MB and MV still remained 95% after six adsorption-desorption cycles. Thus, PFPCS exhibited favorable reusability for the removal of cationic dyes from aqueous solution.

#### Adsorption Mechanism

It is essential to understand the adsorption mechanism of PFPCS for cationic dyes. Considering zeta potentials at different pH values and the effect of solution pH on adsorption capacity, it was presumed that the electrostatic attraction between negatively charged surfaces of PFPCS and positively charged cationic dyes could dominate the selective adsorption for cationic dyes. However, the adsorption capacity of MB and MV increased with the increase in solution pH, demonstrating that, besides electrostatic attraction, other mechanisms also participated in the adsorption process. The Figure 9B shows the FT-IR spectra of PFPCS after adsorption. The new peak at 1,600 cm^−1^ was attributable to aromatic ring stretching vibration of MB and MV after adsorption, indicating the successful adsorption of MB and MV dyes. And the −OH vibration at 3,432 cm^−1^ shifted to 3,442 cm^−1^, which was related to the hydrogen bonding interactions between the PFPCS and the dyes. And (Supplementary Figure S4) shows the surface morphologies of PFPCS after adsorption. The morphology of PFPCS change weakly, indicating that PFPCS was stable during the adsorption process. The EDS spectra of PFPCS after adsorption show in (Supplementary Figure S5). After the adsorption, the new N and S elements were observed at 0.25 and 2.31 keV in the PFPCS sample, respectively. The results indicate that MB and MV were successfully adsorbed onto the surface of PFPCS. This mechanism not only enabled PFPCS to exhibit excellent adsorption capacity for anionic dyes but also endowed it with superior selective adsorption toward dye mixtures. The possible adsorption mechanism, including the adsorption process and the interactions between PFPCS and dyes, is schematically illustrated in (Supplementary Figure S6).

## Conclusion

The PFPCS biosorbent was successfully synthesized and used as a superior biosorbent to remove MB and MV from the aqueous solution. The adsorption process was in good agreement with the pseudo-second-order kinetic and Langmuir isothermal models. And the maximum adsorption capacities were 1,775.76 and 3,756.33 mg g^−1^ for MB and MV, respectively. The adsorption of MB and MV on PFPCS reached equilibrium within 20 min. Moreover, PFPCS was selectively able to adsorb MB and MV on PFPCS from dye mixture. In addition, PFPCS still exhibited excellent reusability even after six desorption-adsorption cycles. Thus, PFPCS had great potential application in efficient removal of cationic with the simple fabrication process, low preparation cost and excellent adsorption performance.

## Data Availability

The original contributions presented in the study are included in the article/Supplementary Material, further inquiries can be directed to the corresponding authors.
