# Supplementary material for: Super and Selective Adsorption of Cationic Dyes onto Carboxylate-Modified Passion Fruit Peel Biosorbent
Source: Front Chem. 2021 May 26;9:646492. doi: 10.3389/fchem.2021.646492 (PMC8189421; doi:10.3389/fchem.2021.646492)
Supplement: Supplementary file 2 [file DataSheet1.docx]

Supplementary Material


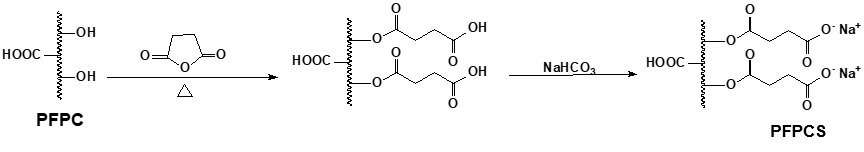


**Scheme S1**. The synthetic route of PFPCS.

**Figure S1**. Derivative thermogravimetric (DTG) curves for PFPC and PFPCS.

**Figure S2**. The N_2_ adsorption-desorption curves of PFPC and PFPCS.


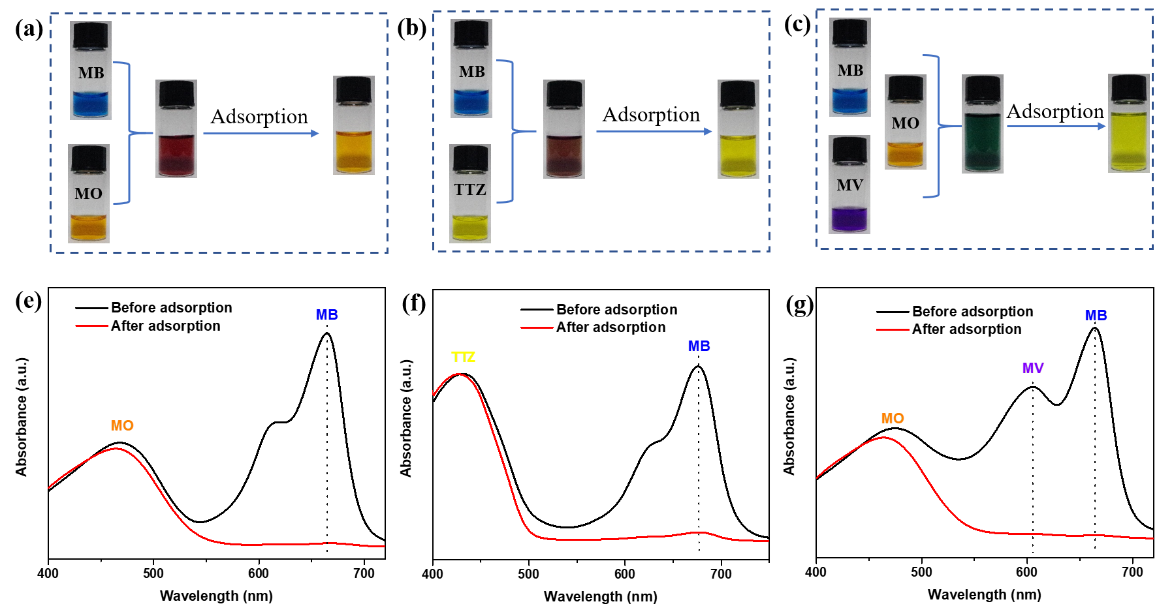


**Figure S3.** Photographs of the dye mixture of MB/TTZ (a), MV/MO (b), and MB/MV/TTZ (c) before and after adding PFPCS for 60 min at pH = 8.0. The initial dye concentration in the mixture is 0.1 mM. The corresponding absorption spectra of the dye mixture of MV/MO (d), MV/TTZ (e), and MB/MV/TTZ (f) before and after adsorption by PFPCS.


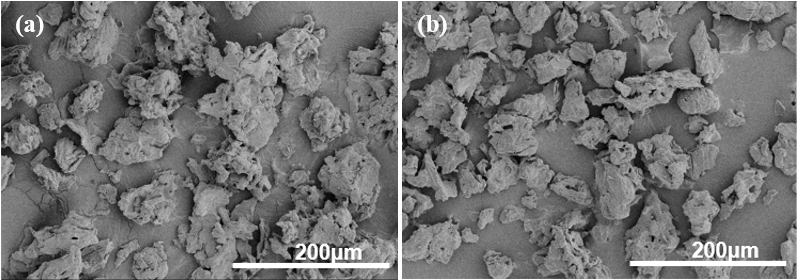


**Figure S4**. The SEM images of MB-adsorbed PFPC (**a**) and MV-adsorbed PFPCS (**b**).

**Figure S5**. Energy-dispersive X-ray spectroscopy (EDS) spectra of the PFPCS, MB-adsorbed PFPCS and MV-adsorbed PFPCS.


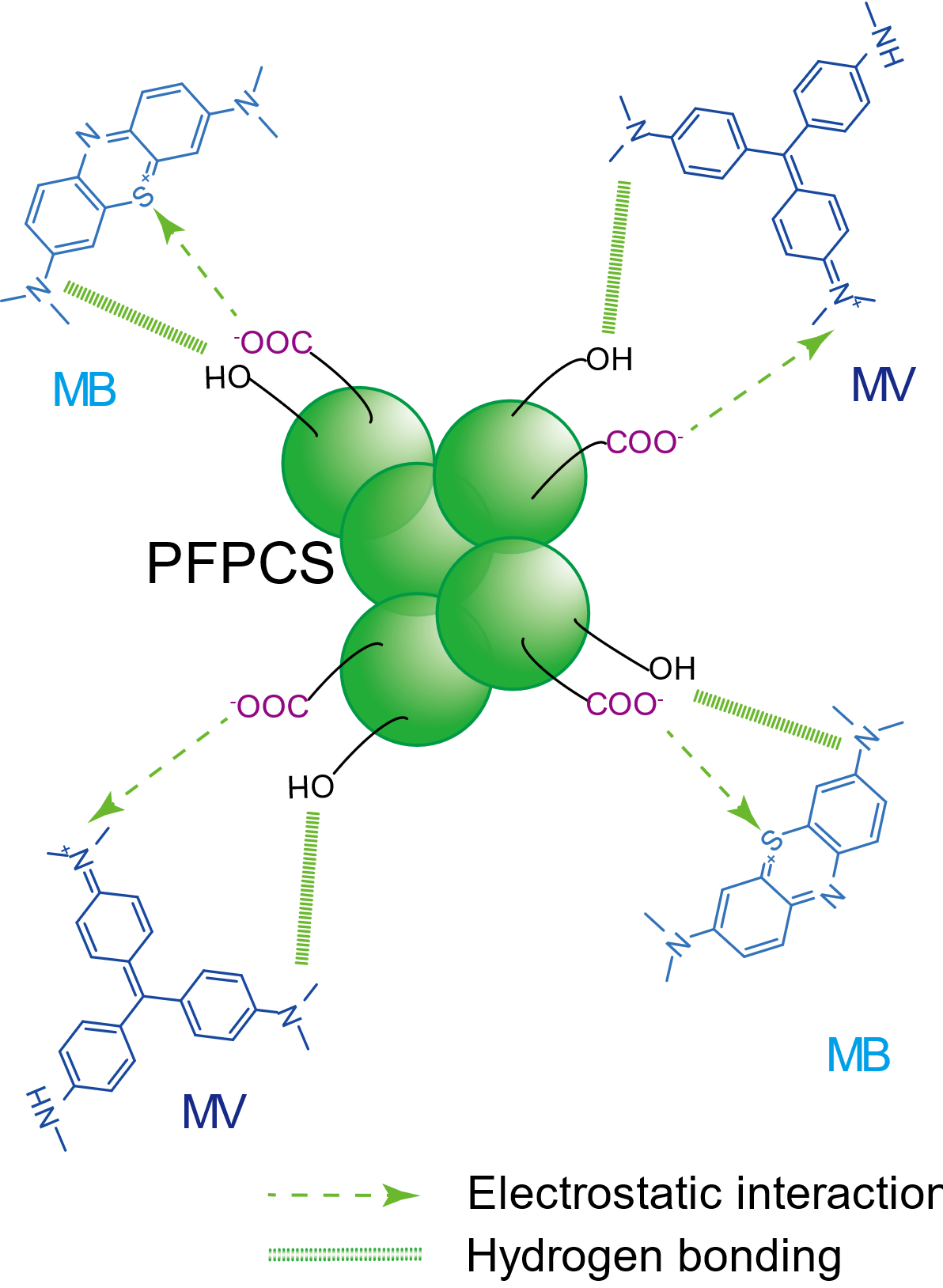


**Figure S6.** Schematic illustration of the adsorption mechanism of PFPCS for cationic dyes.

**Table S1**. Structure and characteristics of the used dyes.

| Dyes | Chemical structure | λ_max_ (nm) | Molecular weight (g·mol^-1^) |
| --- | --- | --- | --- |
| MV |  | 584 | 407.99 |
| MB |  | 664 | 373.90 |
| ART |  | 520 | 604.41 |
| MBR |  | 221 | 461.38 |
| MO |  | 464 | 327.33 |
| TTZ |  | 428 | 534.36 |
